# Author response to comment by Can & Tufan

**DOI:** 10.1007/s40520-024-02910-8

**Published:** 2025-01-13

**Authors:** Mustafa Güldan

**Affiliations:** School of Medicine, Koc University, Istanbul, Turkey

Dear Authors,

Thank you for your thoughtful comments regarding our manuscript. I appreciate the iterative process of feedback, as it represents the core of scientific advancement.

Firstly, the potential confusion raised by your letter may stem from a non-holistic approach to this matter, as both the 6MWD and gait speed are indirect outcomes of decreased muscle strength. Our intention in this manuscript is not to draw distinct boundaries between performance parameters and muscle strength, but rather to present multiple aspects of how bimagrumab affects sarcopenia. We believe that viewing the manuscript from this perspective may help to clarify this issue.

Secondly, while gait speed and the 6MWD are indirect markers of muscle strength and not direct measures, this distinction merits nuanced discussion. We believe this would have been more appropriately addressed in your letter. Slower gait speed, for example, is often indicative of reduced lower-limb strength, particularly in older adults or individuals with mobility impairments. There is robust evidence in the literature demonstrating a correlation between gait speed and leg muscle strength. For instance, in men, stronger knee and hip extensor muscles are positively associated with faster gait speeds, with moderate correlations observed for knee extension (r = 0.415, p = 0.016) and moderate-to-strong correlations for hip extension (r = 0.492, p = 0.004). Among women, stronger lower limb and handgrip muscle forces are significantly associated with faster gait speeds, with notable correlations observed for knee flexion (r = 0.524, p = 0.002), hip extension (r = 0.527, p = 0.002), hip adduction (r = 0.538, p = 0.002), and plantarflexion (r = 0.486, p = 0.006) [1]. Similarly, poor performance in the 6MWD often reflects inadequate lower-limb strength, as walking endurance is dependent on leg muscle condition [2], with additional correlations to upper-limb strength also reported [3].

We hope this response clarifies our position and highlights the broader context in which our findings should be interpreted. Thank you once again for your engagement with our work.

**Kind regards,**

Mustafa Güldan & Mehmet Kanbay

## Data Availability

No datasets were generated or analysed during the current study.
